# Adjuvant statin therapy for oesophageal adenocarcinoma: the STAT‐ROC feasibility study

**DOI:** 10.1002/bjs5.50239

**Published:** 2019-12-14

**Authors:** L. Alexandre, A. B. Clark, S. Walton, M. P. Lewis, B. Kumar, E. C. Cheong, H. Warren, S. S. Kadirkamanathan, S. L. Parsons, S. M. Dresner, E. Sims, M. Jones, M. Hammond, M. Flather, Y. K. Loke, A. M. Swart, A. R. Hart

**Affiliations:** ^1^ Norwich Medical School, University of East Anglia Norwich UK; ^2^ Cancer Research Team and Norwich UK; ^3^ Department of General Surgery Norfolk and Norwich University Hospital NHS Foundation Trust Norwich UK; ^4^ Department of General Surgery Queen Elizabeth Hospital King's Lynn UK; ^5^ Department of General Surgery, Broomfield Hospital Mid Essex Hospital Services NHS Trust Chelmsford UK; ^6^ Department of Surgery, Nottingham City Hospital Nottingham University Hospitals NHS Trust Nottingham UK; ^7^ Department of General Surgery James Cook University Hospital Middlesbrough UK

## Abstract

**Background:**

Statins inhibit proliferative signalling in oesophageal adenocarcinoma (OAC) and their use is associated with better survival in observational studies. The present study was undertaken to examine the feasibility of assessing adjuvant statin therapy in patients with operable OAC in a phase III RCT.

**Methods:**

For this multicentre, double‐blind, parallel‐group, randomized, placebo‐controlled feasibility trial, adults with OAC (including Siewert I–II lesions) who had undergone oesophagectomy were centrally allocated (1 : 1) to simvastatin 40 mg or matching placebo by block randomization, stratified by centre. Participants, clinicians and investigators were blinded to treatment allocation. Patients received treatment for up to 1 year. Feasibility outcomes were recruitment, retention, drug absorption, adherence, safety, quality of life, generalizability and survival.

**Results:**

A total of 120 patients were assessed for eligibility at four centres, of whom 32 (26·7 per cent) were randomized, 16 in each group. Seven patients withdrew. Participants allocated to simvastatin had lower low‐density lipoprotein cholesterol levels by 3 months (adjusted mean difference −0·83 (95 per cent c.i. −1·4 to −0·22) mmol/l; *P* = 0·009). Median adherence to medication was greater than 90 per cent between 3 and 12 months' follow‐up. Adverse events were similar between the groups. Quality‐of‐life data were complete for 98·3 per cent of questionnaire items. Cardiovascular disease, diabetes and aspirin use were more prevalent in the non‐randomized group, whereas tumour site, stage and grade were similar between groups. Survival estimates were imprecise.

**Conclusion:**

This RCT supports the conduct and informs the design considerations for a future phase III trial of adjuvant statin therapy in patients with OAC. Registration number: ISRCTN98060456 (http://www.isrctn/com).

## Graphical abstract

After oesophagectomy for oesophageal adenocarcinoma (OAC), the 5‐year survival rate is currently only 30 per cent. There is strong observational evidence that statin use is associated with reduced all‐cause and cancer‐specific mortality in patients with OAC. This multicentre, double‐blind, parallel‐group, randomized, placebo‐controlled trial has established the feasibility of a future phase III trial of adjuvant statin therapy in patients treated with curative intent for invasive OAC.

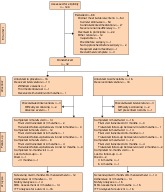

Justification for a full trial

## Abstract (es)

**Antecedentes:**

Las estatinas inhiben las señalizaciones proliferativas en el adenocarcinoma de esófago (*oesophageal adenocarcinoma*, OAC) y su uso se asocia con mejor supervivencia en estudios observacionales. El presente estudio se llevó a cabo para examinar la viabilidad de evaluar el tratamiento adyuvante con estatinas en pacientes con OAC operable en un ensayo aleatorizado y controlado de fase III.

**Métodos:**

En este ensayo de viabilidad controlado por placebo, aleatorizado, de grupos paralelos, doble ciego y multicéntrico, los pacientes adultos con OAC (incluyendo lesiones Siewert I/II) que fueron sometidos a esofaguectomía se asignaron de forma centralizada (1:1) a tratamiento con simvastatina 40 mg o placebo equivalente mediante aleatorización en bloques, estratificados por centro. Los participantes, los clínicos y los investigadores desconocían la asignación del tratamiento. Los pacientes recibieron el tratamiento hasta un año. Los resultados de viabilidad fueron reclutamiento, retención, absorción del fármaco, adherencia, seguridad, calidad de vida, generalización, y supervivencia.

**Resultados:**

Un total de 120 pacientes fueron evaluados para elegibilidad en 4 centros, de los cuales 32 (26,7%) fueron aleatorizados, 16 en cada grupo. Siete pacientes abandonaron el ensayo. Los pacientes asignados a tratamiento con simvastatina tenían niveles de colesterol LDL más bajos a los 3 meses (diferencia media ajustada, −0,83 mmol/L, i.c. del 95% −1,4 a −0,22, *P* = 0,009). La mediana de la adherencia a la medicación fue mayor del 90% entre los 3‐12 meses de seguimiento. Los eventos adversos fueron similares entre los grupos. Los datos de calidad de vida estaban completos en el 98,3% de las preguntas del cuestionario. Enfermedad cardiovascular, diabetes y uso de aspirina eran más prevalentes en el grupo no aleatorizado, mientras que la localización del tumor, el estadio y el grado fueron similares entre los grupos. Las estimaciones de supervivencia fueron imprecisas.

**Conclusión:**

Este RCT apoya la realización e informa de las consideraciones de diseño para un futuro ensayo de fase III de tratamiento adyuvante con estatinas en pacientes con OAC.

## Introduction

Oesophageal adenocarcinoma (OAC) is an aggressive malignancy with a poor prognosis^1^. Approximately 40 per cent of patients with OAC are treated with curative intent by oesophagectomy^2^, with or without perioperative chemotherapy or chemoradiotherapy^3^. Despite these high‐risk interventions^2^, the 5‐year survival rate is still only 30 per cent^4^, with mortality attributable mainly to recurrent disease^5^. Currently, there are no longer‐term evidence‐based interventions after curative surgical resection to prevent cancer recurrence and reduce mortality.

There is growing experimental and observational evidence that statins (3‐hydroxy‐3‐methylglutaryl coenzyme A reductase inhibitors) could be effective agents in the adjuvant setting^6^. Preclinical studies^7^, ^8^, ^9^, ^10^ have demonstrated antiproliferative, proapoptotic and antimetastatic effects of statins in OAC. Gain‐of‐function mutations in *p53*, the most commonly mutated gene in OAC^11^, have been shown to upregulate transcription of mevalonate pathway enzymes that sustain malignant proliferation^12^, ^13^, suggesting that this pathway may be a therapeutic target. A recent systematic review^14^ of observational studies, which included 95 cohorts with over a million patients with cancer at numerous primary sites, demonstrated that statin use after diagnosis was associated with significant reductions in all‐cause mortality (pooled hazard ratio (HR) 0·65, 95 per cent c.i. 0·60 to 0·72). Large independent population‐based cohort studies^15^, ^16^, ^17^ of patients with OAC demonstrated that postdiagnostic statin use was associated with significant reductions in all‐cause and oesophageal cancer‐specific mortality. Importantly, the association remains robust when accounting for cancer stage, immortal‐time bias and reverse‐causation bias.

Malignant recurrence of OAC occurs despite careful exclusion of overt disseminated disease at diagnosis with preoperative staging modalities, implying the presence of subclinical micrometastatic cancer at the time of oesophagectomy^18^. This group of patients has been selected to investigate the adjuvant effects of statins as they have minimal disease burden, yet substantial risk of recurrent disease^19^. Statins represent attractive adjuvant agents to investigate in this setting as they are easily administered, inexpensive and well tolerated, with an excellent safety profile^20^, ^21^, ^22^, ^23^. To establish the efficacy of statins as adjuvant agents for OAC, a definitive phase III RCT is required. However, uncertainties exist surrounding its feasibility and conduct, particularly in the context of investigating a repurposed drug for an adjuvant indication. The aim of this study was to determine the feasibility of a phase III RCT of adjuvant statin therapy in patients following oesophagectomy for OAC. The objectives were to determine: recruitment and retention rates; generalizability of randomized to non‐randomized patients; drug absorption; treatment adherence; a preliminary safety profile of simvastatin in this patient population; completion rates of questionnaires and exploratory comparisons of quality of life; and to estimate treatment efficacy on disease‐free and overall survival.

## Methods

This feasibility study is reported in accordance with the extension to the CONSORT 2010 statement for randomized pilot and feasibility trials^24^. The final study protocol is available on request. Ethical approval was received from National Research Ethics Service Committee South Central, Oxford B (reference 14/SC/0247). The trial received Medicines and Healthcare products Regulatory Agency approval (reference 13630/005/001‐0002). The trial was registered with the European Clinical Trials Database (EudraCT number 2014‐001318‐24) and the ISRCTN registry (ISRCTN98060456).

### Trial design and participants

The STATin therapy in the prevention of post‐operative Recurrence of Oesophageal adenoCarcinoma (STAT‐ROC) feasibility study is a multicentre, double‐blind, parallel‐group, randomized, placebo‐controlled trial of the feasibility of assessing adjuvant statin therapy in a phase III trial. An amendment to the protocol was approved to ensure recruitment was sufficient to meet the minimum target of 24 patients and improve study generalizability. The study was allowed to expand from one to four sites, and to extend recruitment from 31 October 2015 to 31 July 2016. Given that the recruitment period was extended and the date for the end of the trial fixed (31 October 2016), follow‐up was truncated for participants recruited after 1 November 2015, which enabled at least 3 months' follow‐up to determine drug absorption.

Adults with OAC (including adenocarcinoma of the gastro‐oesophageal junction, Siewert I–II lesions) due to undergo curative resection were eligible. Patients were excluded if they were already prescribed a statin or had a contraindication to statin therapy. The eligibility criteria are listed in full in *Table* *S1* (supporting information).

### Randomization and masking

Patients were assigned randomly to receive simvastatin 40 mg or matched placebo in a 1 : 1 ratio. A computer‐generated randomization code (generated by Ipswich Pharmacy Manufacturing Unit) was used to randomize participants in blocks of four to six, stratified by site. The randomization code stipulated the treatment allocation according to a sequentially ordered four‐digit subject number. Identical sealed medication bottles were labelled individually with corresponding subject numbers to preserve allocation concealment. Participants were allocated a subject number sequentially in the order they passed the baseline assessment. An interactive web response system, with password access limited to registered investigators, allocated the subject numbers serially to recruited patients. To preserve blinding, Ipswich Pharmacy Manufacturing Unit produced identical active and placebo capsules. Blood samples to measure low‐density lipoprotein (LDL) cholesterol were frozen and analysed after completion of the trial. Participants, their healthcare providers, data collectors and outcome adjudicators were all blinded to treatment allocation.

### Procedures

Participants were identified at local upper gastrointestinal cancer multidisciplinary team meetings at each site. During the recruitment period, a retrospective pseudoanonymous review of all patients with OAC due to undergo curative surgery (regardless of any exclusion criteria present) was conducted. This generated a reference population against which generalizability was assessed for the randomized study population.

Potentially eligible patients were approached to consider participation in the preoperative period during an outpatient clinic appointment with their surgeon or oncologist. They were issued an invitation letter and a participant information sheet. A member of the research team saw them at a screening visit before surgery, which usually coincided with the day of their preoperative assessment. During this visit, participants were screened to determine eligibility, informed written consent was obtained, clinical data were collected, blood was taken for tests for safety (thyroid function tests, liver function tests (LFTs), creatine kinase and creatinine estimation) and research (LDL cholesterol)^5^, quality‐of‐life questionnaires (European Organisation for Research and Treatment of Cancer (EORTC) QLQ‐C30 and disease‐specific oesophagogastric QLQ‐OG25 module questionnaires) were completed, and feasibility study acceptance/declined questionnaires (as appropriate) were completed. The research team wrote to the patient's general practitioner to confirm whether they were due to be prescribed a statin.

Randomization took place for consenting participants who satisfied the screening and eligibility assessments, and who were due to be discharged after surgery. Participants were prescribed trial medication once‐daily to start from the date of discharge.

Participants were followed up at 3, 6, 9 and 12 months after discharge. Follow‐up assessments were scheduled to coincide with hospital appointments as part of the participant's usual care. Follow‐up visits included: confirmation of any clinical contraindication to receiving trial medication; assessment of adverse events; drug safety assessments (LFTs at 3 and 12 months, and creatine kinase estimation if muscle symptoms developed and the trial medication was considered likely to be causal); quality‐of‐life questionnaires (as above); pill counts; blood tests for non‐fasting LDL cholesterol levels; medical notes review to determine disease outcomes, including cancer recurrence; physical examination for evidence of recurrence if not already diagnosed; and dispensing of trial drugs at 3, 6 and 9 months from discharge after surgery. Participants received trial medication for up to 1 year.

The trial steering and safety committees were consulted every 6 months for the duration of the trial.

### Statistical analysis

A statistical analysis plan was finalized and approved by the trial steering committee before blinding was broken and analysis undertaken. The analyst was blind to group allocation until analysis was complete. All eight outcomes were viewed with equal primacy.

#### *Recruitment*

Recruitment was defined as the randomization of a trial participant. Three aspects of recruitment were calculated: the proportion of participants randomized from all those who were assessed for eligibility (the denominator was those with OAC due to have surgery); the proportion of participants randomized from those who met all eligibility criteria (except for whether or not they were willing to consent); and the number of participants randomized per month per centre.

#### *Retention*

Retention was defined as the date of withdrawal, which included both complete withdrawal from the trial and withdrawal of treatment but still undergoing active follow‐up, censored for recurrence or death. Kaplan–Meier survival curves were plotted for the randomized population and by treatment group. Differences between groups were determined with the log rank test.

#### *Absorption*

The primary outcome was change in non‐fasting LDL cholesterol at 3 months after discharge, adjusted for LDL cholesterol measured at screening in the intention‐to‐treat (ITT) population. Adjusted and unadjusted mean differences in LDL cholesterol, measured between treatment groups for 3–12 months, were tabulated. Unadjusted comparisons were made using Student's *t*‐test, and adjusted comparisons were conducted using analysis of co‐variance (ANCOVA). Sensitivity analyses using the method above were repeated for the per‐protocol population, defined for this outcome as participants who adhered to least 80 per cent of dispensed medications in the preceding 3 months.

#### *Generalizability*

Demographic and clinical characteristics were compared between randomized and non‐randomized patients assessed for eligibility (who otherwise met the inclusion criteria). Categorical data were compared using the χ^2^ test or Fisher's exact test as appropriate, and continuous data using the two‐sample *t* test or Mann–Whitney *U* test as dictated by the distribution.

#### *Adherence*

Adherence was defined as the proportion of medication consumed in the 3 months preceding the research visit (determined using pill counts as the actual number consumed divided by the expected number consumed (days elapsed between previous dispensation and subsequent visit)). Adequate adherence was defined as administration of at least 80 per cent of trial medication in this 3‐month period. Estimates for adherence exceeding 105 per cent for the preceding 3 months were considered implausible and ignored (as were estimates for subsequent visits that were reliant on these).

#### *Safety*

All reported adverse events were summarized according to treatment received and tabulated with frequencies (for the number of individuals with 1 or more adverse events) according to category of adverse event and worst grade experienced using Common Terminology Criteria for Adverse Events v4.0^25^. Safety analyses were restricted to the trial population that successfully administered at least one dose of trial medication. To address a protocol violation, whereby a patient allocated to placebo was dispensed simvastatin in error at 6 months, their safety data were contributed to both groups (the placebo group until the violation, and the simvastatin group thereafter).

#### *Quality of life*

Compliance for completing quality‐of‐life questionnaire items was tabulated for each study visit. Items on both the EORTC QLQ‐C30 and QLQ‐OG25 were scored and scaled, and missing values imputed in line with the EORTC manual^26^. Differences in mean scores between groups were adjusted for values observed at screening using ANCOVA.

#### *Exploratory survival comparisons*

Overall survival was defined as time elapsed from discharge from hospital to death from any cause. Disease‐free survival was defined as the time elapsed from discharge to the first time point at which recurrence or death occurred. Kaplan–Meier survival curves with Cox proportional hazards modelling compared treatment groups. All analyses were performed with STATA® version 13 (StataCorp, College Station, Texas, USA).

#### *Sample size*

A sample size of 24 was the minimum recruitment target for the trial (gains in the precision of the mean difference for outcomes measured on a continuous scale for participants randomized in a 1 : 1 ratio are small above this number) and was expected to satisfy assessment of feasibility outcomes measured on a continuous scale^27^. A sample of 22 participants (11 per arm) had 80 per cent power at the 5 per cent level to detect a difference of 1 mmol/l in LDL cholesterol concentration, assuming a standard deviation of 0·8^28^.

## Results

**Figure 1:**
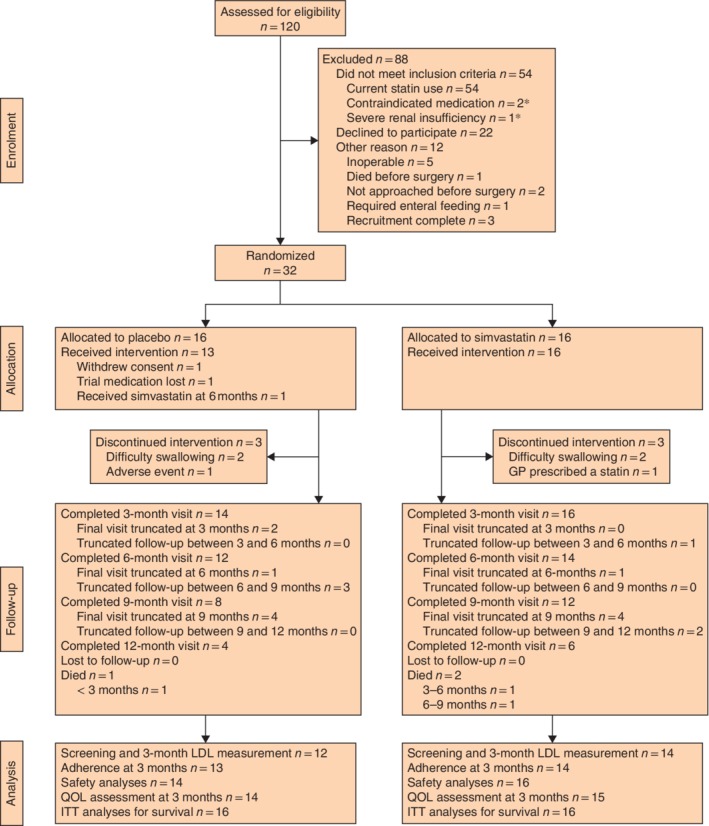
Trial profile *In addition to being current statin users, three patients also met the other exclusion criteria listed. GP, general practitioner; LDL, low‐density lipoprotein; QOL, quality of life; ITT, intention to treat.

Between 21 October 2014 and 22 July 2016, 120 patients with OAC who were due to undergo potentially curative surgery in four UK oesophagogastric centres (Norwich, Chelmsford, Nottingham and Middlesbrough) were assessed for eligibility (*Fig*. 1). In total 88 were excluded, of whom 54 were ineligible as they were current statin users. Of the 120 patients assessed for eligibility, 32 (26·7 (95 per cent c.i. 19·0 to 35·5) per cent) were randomized equally to simvastatin or placebo. The proportion of participants randomized from those who met all eligibility criteria (except whether or not they were willing to consent) was 59 (45·0 to 72·4) per cent (32 of 54). Of the three sites that randomized patients, the recruitment rates (per month) in descending order were 1·31 (95 per cent c.i. 0·49 to 2·79), 1·16 (0·73 to 1·76) and 0·54 (0·18 to 1·24), and the cumulative recruitment rate (per month) for these sites was 3·01 (2·59 to 3·48).

### Baseline characteristics

Participant characteristics were generally well balanced between treatment groups (*Table* 1). Mean(s.d.) age was 62·7(12·3) years in the placebo group and 66·6(8·7) years in the simvastatin group. Thirteen participants in the placebo group and 12 in the simvastatin group were men. Fifteen patients in each treatment group received neoadjuvant chemotherapy. The majority of patients in both groups received trial medication on the day they were randomized.

**Table 1:** Baseline characteristics of randomized participants

	Placebo (*n* = 16)	Simvastatin (*n* = 16)
Age at randomization (years)*	62·7(12·3)	66·6(8·7)
Time from diagnosis to randomization (days)*	153·4(31·8)	155(40·8)
Time from randomization to receiving trial medication (days)	0 (0–1)	0 (0–0)
Sex ratio (M : F)	13 : 3	12 : 4
Smoking status		
Current smoker	1	2
Ex‐smoker	10	11
Never smoked	5	3
BMI (kg/m^2^)*	26·2(4·1)	26·6(4·7)
Co‐morbid condition		
Cardiovascular	0	1
Diabetes	0	0
Charlson co‐morbidity index†		
0	15	14
1	1	2
Perioperative aspirin use	0	0
ECOG performance status		
0	16	13
1	0	2
2	0	1
LDL cholesterol (mmol/l)*	3·51(0·89)	3·73(0·92)
Tumour site		
Oesophagus	7	5
Siewert I	2	4
Siewert II	7	7
Tumour grade		
Gx	2	1
G1	0	0
G2	5	8
G3	9	6
G4	0	1
cT category		
2	0	1
3	16	12
4	0	1
4a	0	2
cN category		
0	2	5
1	9	6
2	4	4
3	1	1
Neoadjuvant chemotherapy		
Yes	15	15
No	1	1
Preoperative radiotherapy		
Yes	0	1
No	16	15
Oesophagectomy		
Open	4	2
Hybrid	9	10
Minimally invasive	3	4
Lymph node yield	26 (19–42)	22 (25–35)
Positive lymph nodes	1·5 (0–4·5)	1 (0–3)
Vascular invasion		
Yes	9	5
No	7	11
Margin status		
R1	4	3
R0	12	13
Postoperative length of stay (days)	10 (6–13)	9 (6–12)
Any postoperative in‐hospital complication	7	6
Global quality‐of‐life score*, ‡	68(20)	73(10)

Values in parentheses are interquartile ranges unless indicated otherwise;

* values are mean(s.d.).

† Modified Charlson co‐morbidity index (excludes solid tumours).

‡ A high score suggests a high level of functioning. ECOG, Eastern Cooperative Oncology Group; LDL, low‐density lipoprotein.

### Retention

**Figure 2:**
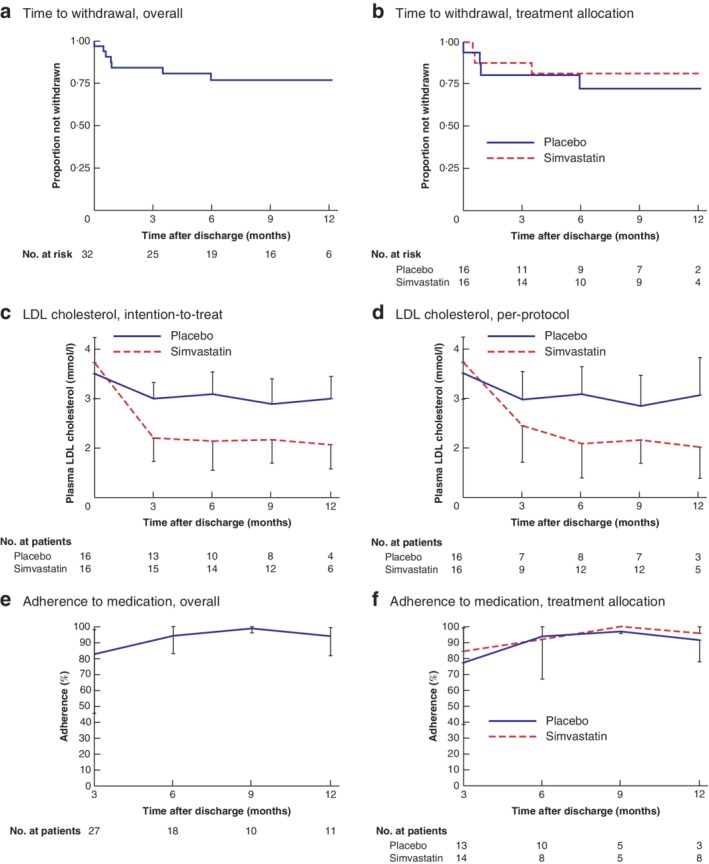
**Kaplan–Meier analysis of time to withdrawal, low‐density lipoprotein cholesterol during follow‐up, and adherence to trial medication** **a,b** Time to withdrawal overall (**a**) and according to treatment allocation (**b**). **c,d** Mean plasma low‐density lipoprotein (LDL) cholesterol levels during follow‐up according to treatment allocation for intention‐to‐treat (**c**) and per‐protocol (**d**) populations. Half error bars span from the mean to the upper or lower limit of the 95 per cent confidence interval. **e,f** Median percentage adherence to trial medication overall (**e**) and according to treatment allocation (**f**), calculated at each trial visit for the preceding 3 months. Half error bars span from the median to the upper or lower limit of the interquartile range.

Retention did not differ between treatment groups (*P* = 0·630, log rank test). In total, seven patients withdrew from the trial: four reported difficulty swallowing the trial medication, one withdrew consent, one was prescribed a non‐trial statin during follow‐up by their general practitioner, and one developed transaminitis (*Fig*. 2 *a,b*; *Table* *S2*, supporting information). Aside from two withdrawals between 3 and 6 months, all other withdrawals happened within 27 days of randomization. The overall annual rate of withdrawal per person was 0·36 (95 per cent c.i. 0·17 to 0·76). The highest rate was observed in the first 3 months (0·74, 0·31 to 1·77), before falling between 3 and 6 months to 0·36 (0·09 to 1·46); thereafter there were no further losses to follow‐up (*Table* *S3*, supporting information).

### Generalizability

No significant differences were found between non‐randomized and randomized patient populations for age at diagnosis, sex, smoking status, BMI, tumour site, tumour grade, clinical stage or preoperative radiotherapy (*Table* *S4*, supporting information). As expected, there were significant differences between treatment groups for cardiovascular disease, diabetes and aspirin use, which were all more prevalent in the non‐randomized group. Furthermore, a significantly higher proportion in the randomized group had better performance status, and underwent neoadjuvant chemotherapy.

### Absorption

Compared with placebo users, patients allocated to simvastatin had a significantly lower mean difference in LDL cholesterol by 3 months, adjusted for values at screening (0·83 (95 per cent c.i. −1·40 to 0·22) mmol/l; *P* = 0·009) (*Table* 2 and *Fig*. 2 *c,d*), equivalent to a 27·6 per cent proportionate reduction. Similar reductions in LDL cholesterol persisted for the maximum duration of follow‐up (12 months).

**Table 2:** Comparison of non‐fasting plasma low‐density lipoprotein cholesterol by treatment group

	Placebo		Simvastatin					
	*n*	LDL (mmol/l)*	*n*	LDL (mmol/l)*	Unadjusted mean difference	*P* ‡	Adjusted mean difference†	*P* ‡
**Intention‐to‐treat**								
3‐month visit	13	3·00(0·54)	15	2·20(0·85)	−0·80 (−1·36 to −0·24)	0·007	–0·83 (–1·40 to –0·22)	0·009
6‐month visit	10	3·09(0·63)	14	2·14(1·01)	−0·95 (−1·71 to −0·20)	0·016	–1·23 (–1·85 to –0·40)	0·004
9‐month visit	8	2·89(0·61)	12	2·17(0·74)	−0·72 (−1·39 to −0·05)	0·036	–0·79 (–1·47 to –0·11)	0·025
12‐month visit	4	3·00(0·28)	6	2·07(0·47)	−0·93 (−1·54 to −0·33)	0·008	–0·99 (–1·58 to –0·40)	0·007
**Per‐protocol**								
3‐month visit	7	3·00(0·60)	9	2·46(0·96)	−0·53 (–1·42 to 0·36)	0·224	–0·49 (–1·47 to 0·49)	0·300
6‐month visit	8	3·09(0·66)	12	2·09(1·09)	–1·00 (–1·91 to –0·09)	0·034	–1·16 (–2·01 to –0·32)	0·010
9‐month visit	7	2·86(0·66)	12	2·17(0·74)	–0·69 (–1·41 to 0·03)	0·058	–0·74 (–1·47 to –0·00)	0·049
12‐month visit	3	3·07(0·31)	5	2·02(0·51)	–1·05 (–1·85 to –0·24)	0·019	–1·16 (–1·96 to –0·35)	0·016

Values in parentheses are 95 per cent confidence intervals unless indicated otherwise;

* values are mean(s.d.). Analysis by ANCOVA.

† Adjusted for screening values. A negative difference implies that patients taking simvastatin have a lower low‐density lipoprotein (LDL) cholesterol level than those on placebo.

‡ Student's *t*‐test and ¶ calculated by ANCOVA.

### Adherence

Overall adherence was lowest in the first 3 months of treatment (median adherence 83 (i.q.r. 45–98) per cent) before improving at subsequent visits at 6 months (median 94 (83–100) per cent), 9 months (median 99 (96–100) per cent) and 12 months (median 94 (82–99) per cent) (*Table* 3 and *Fig*. 2 *e,f*). Adherence was similar between the two groups.

**Table 3:** Adherence to trial medication during follow‐up

	Placebo	Simvastatin	Overall
**3‐month visit**			
No. of patients	13	14	27
No. with adherence ≥ 80%	6	8	14
Adherence (%)*	77 (38–98)	85 (63–99)	83 (45–98)
**6‐month visit**			
No. of patients	10	8	18
No. with adherence ≥ 80%	8	6	14
Adherence (%)*	94 (90–100)	92 (67–99)	94 (83–100)
**9‐month visit**			
No. of patients	5	5	10
No. with adherence ≥ 80%	5	5	10
Adherence (%)*	97 (96–99)	100 (99–100)	99 (96–100)
**12‐month visit**			
No. of patients	3	5	8
No. with adherence ≥ 80%	2	4	6
Adherence (%)*	92 (78–98)	96 (85–100)	94 (82–99)

* Median (i.q.r.) percentage adherence to at least 80 per cent of trial medication in the preceding 3 months. No implausible values for adherence (greater than 105 per cent) were observed at 3 months. Values from three patients were ignored at 6 months (and thereafter), values from five patients were ignored at 9 months (and thereafter) and none were ignored at 12 months.

### Safety

There were 108 individual adverse events affecting 27 participants (*Table* 4; *Table* *S5*, supporting information). In total 20 (18·5 per cent) were serious adverse events (13 in the placebo and 7 in the simvastatin arm). There were no suspected serious adverse reactions or suspected unexpected serious adverse reactions. Before unblinding, of all individual adverse events 94 were assessed as unrelated to the trial medication, 12 were assessed as unlikely to be related, and two were assessed to be possibly related (grade 3 transaminitis, which was subsequently downgraded to grade 2 in the same patient, who had been allocated placebo). There were similar proportions and severity grading of individual adverse events in each group.

**Table 4:** Adverse events by treatment allocation

	No. with at least 1 adverse event	
CTCAE system organ class	Placebo	Simvastatin
**Blood**	1	0
**Ear**	1	1
**Gastrointestinal**	11	9
**General disorders**	3	5
**Infections**	3	4
**Investigations**	3	2
Transaminitis	1	0
**Metabolism and nutrition**	0	1
**Musculoskeletal**	1	5
Myalgia	1	2
**Neoplasms**	1	0
**Nervous system**	2	3
**Psychiatric**	2	2
**Renal and urinary**	1	1
**Respiratory**	4	2
**Skin**	1	1
**Vascular**	2	1
**Any**	13	14

CTCAE, Common Terminology Criteria for Adverse Events.

### Quality of life

Overall completion of questionnaire items was 98·3 per cent (6278 of 6385); thus, 107 values (1·7 per cent) were imputed. Overall, adjusted differences between groups for QLQ‐C30 function scores and QLQ‐OG25 symptoms scales were small (*Table* *S6*, *Figs* *S1* and *S2*, supporting information).

### Survival

During 22·9 person‐years of follow‐up, four participants developed distal recurrent disease (2 in each group); of these, three patients died (1 in the placebo and 2 in the simvastatin group). Median overall and disease‐free survival were not reached. There was no significant difference between groups for overall (HR 1·56, 95 per cent c.i. 0·14 to 17·28; *P* = 0·716) or disease‐free (HR 0·78, 0·11 to 5·61; *P* = 0·807) survival (*Fig*. *S3*, supporting information).

## Discussion

This multicentre, double‐blind, parallel‐group, placebo‐controlled trial has reported outcomes for 32 participants randomized to simvastatin 40 mg or placebo to determine the feasibility of adjuvant statin therapy in patients with operable OAC in a future phase III trial.

The study has demonstrated that patients were willing to enter the trial, and their consultants were willing to recruit them. The proportion of patients randomized from those assessed for eligibility and from those meeting inclusion criteria was favourable, and informs the recruitment of centres for a future RCT. Assuming 4 years of recruitment, with 3 years' follow‐up after the last randomization, an absolute mortality difference of 7 per cent by 3 years (HR 0·80), 80 per cent power at the 5 per cent significance level, and accounting for attrition and contamination of the exposure, would require 976 patients. Assuming an average recruitment rate of 26·7 per cent (derived from this feasibility study, which accounts for prevalent statin use) of patients undergoing oesophagectomy for OAC, with participation of at least 25 UK oesophagogastric cancer centres, complete recruitment within 4 years is feasible. Rates of withdrawal were highest in the first 3 months of follow‐up, contributed to mainly by difficulties in swallowing trial medication. Participants who continued to participate from 1 month after discharge were more likely to be retained. These data provide strong impetus to manufacture smaller, bespoke, more easily swallowed (and ideally suitable for crushing) study medication for a future trial. The early dropouts observed provide justification for the implementation of a run‐in period before randomization in a future trial. As a result of these planned adaptations, it is expected the withdrawal rates in a future phase III RCT would be lower than that observed in this feasibility study.

Comparisons between randomized and non‐randomized groups provide strong evidence for systematic differences between groups for the prevalence of cardiovascular disease, diabetes and aspirin use. This is expected as the trial excluded users of statins, indicated in patients with these conditions, often with shared indications for aspirin use.

A significant reduction in non‐fasting LDL cholesterol between randomized treatment groups at 3 months in the ITT population provides good evidence to infer that statins are absorbed sufficiently to produce a pharmacodynamic effect. This is important to demonstrate, as patients are vagotomized during oesophagectomy, affecting gastrointestinal transit, which could hypothetically impair drug absorption. Reductions in LDL cholesterol were consistent with those observed in the Medical Research Council–British Heart Foundation Heart Protection Study^28^ (mean(s.e.) difference overall −1·0(0·02) mmol/l). Longer‐term statin absorption over the course of the trial was also confirmed.

Adherence, determined using pill counts, was poorest in the first 3 months, but was greatly improved thereafter. At least three‐quarters of the cohort adhered to at least 80 per cent of the trial medication from 3 months. Adherence data are applicable only to the first year of treatment. Adherence was similar between treatment groups. Furthermore, interpretation should also consider the known limitations of pill counts, as they can overestimate adherence^29^. Nevertheless, the data from pill counts taken together with the comparison of LDL cholesterol between groups would suggest that adherence was sufficient to support a future trial.

There was no evidence to suggest an adverse safety profile in this patient population with statin use, either in terms of the absolute numbers of adverse events or in terms of their severity. There is no plausible reason to suggest the adverse event profile should be different in this cohort, and there are no known interactions with current chemotherapy regimens and statins, and no evidence of excess of harm when co‐administered in the trial setting^30^, ^31^. Completion of both the QLQ‐C30 and QLQ‐OG25 questionnaires was high overall (98·3 per cent) and at each follow‐up visit, suggesting the feasibility of assessing quality of life in a future phase III RCT. Adjusted differences between groups were small and not clinically significant (a value of 8 points difference has previously been deemed to be of clinical importance)^32^. In line with guidance on the conduct of feasibility studies, this study was not powered to test efficacy. Estimates of overall and disease‐free survival were imprecise with contradictory point estimates, precluding their use in determining an effect size for a future trial.

This is the first RCT to determine the feasibility of assessing postoperative statin therapy in patients with OAC in a future phase III study. The effect of simvastatin 80 mg has been assessed previously in patients undergoing oesophagectomy in a single‐centre RCT^33^. The aim of that study was to determine the effect of perioperative simvastatin 80 mg (*versus* placebo) on pulmonary dead space to determine the potential of high‐dose statin therapy in preventing acute lung injury. Similar to the present study, the prevalence of statin use was high (31 of 63 patients excluded (49 per cent) were prevalent statin users). Published trials (3 of which were phase III and have been summarized previously^34^) have assessed the effect of allocation to statins on mortality outcomes in patients with solid tumours^30^, ^31^, ^35^, ^36^, ^37^, ^38^, ^39^, ^40^, ^41^ including gastro‐oesophageal^30^, ^31^, colorectal^35^, pancreatic^36^, hepatocellular^37^ and lung^38^, ^40^, ^41^. The largest RCT to date, LUNGSTAR^40^, of 846 patients with small‐cell lung cancer, demonstrated no improvement with pravastatin 40 mg on overall survival (HR 1·01, 95 per cent c.i. 0·88 to 1·16). In 244 patients with metastatic gastro‐oesophageal junction or gastric adenocarcinoma, simvastatin 40 mg in addition to palliative chemotherapy did not improve overall survival. However, it is difficult to draw comparison with previous trials as they all recruited patients with known metastatic disease, and the potential benefits of statins are likely greatest in the adjuvant setting. Of relevance, there was no evidence to suggest a clinically significant increase in toxicity with statin allocation.

Add‐Aspirin, a phase III RCT of adjuvant aspirin therapy in four disease cohorts (gastro‐oesophageal, breast, prostate and colorectal cancer) is currently recruiting^42^. Patients with adenocarcinoma, adenosquamous carcinoma or squamous cell carcinoma of the oesophagus, gastro‐oesophageal junction or stomach are eligible for inclusion in the gastro‐oesophageal cohort, and hence the inclusion criteria are broader than for STAT‐ROC. Add‐Aspirin demonstrates the appetite of funders, investigators, clinicians and patients for investigating repurposed medication as adjuvant cancer therapy. One patient was successfully co‐enrolled into both trials.

This study has a number of strengths. It was possible to assess the ‘real‐world’ feasibility of a future phase III RCT in the setting of a multicentre trial, to provide valid estimates of feasibility parameters. The trial has established the prevalence of statin use in the target trial population, a notable risk to study feasibility. These data are informative in assessing trial feasibility to enable planning of expected recruitment. This trial has provided valuable information for devising strategies to improve retention in a future trial, particularly regarding the manufacture of smaller trial medication that can be easily swallowed and ideally crushed. It was also possible to establish that trial procedures were acceptable at different sites to clinicians, research staff and patients.

This study has a number of limitations. Despite use of the smallest available simvastatin tablets and smallest possible gelatine capsules to preserve blinding, the trial medications were relatively large (measuring 23·3 × 8·53 mm). Of patients who withdrew, difficulty swallowing the capsules was the most commonly cited reason. Although this trial estimated retention, this is unlikely to be applicable to a future trial where use of smaller trial medication would be justified and viable. This makes estimates of retention less certain, necessitating further assumptions for a future trial. Retention in a future trial would be much improved with use of an open‐label run‐in period and smaller trial medication. Feasibility estimates support the conduct and inform the design considerations for a future trial.

## Supporting information


**Table S1.** Eligibility criteria
**Table S2.** Reasons for withdrawal of treatment
**Table S3.** Withdrawal rates for recruited participants, stratified by follow‐up period
**Table S4.** Comparison of demographic and clinical variables between non‐randomized and randomized patient populations
**Table S5.** Adverse events stratified by worst grade experienced and treatment group
**Table S6.** Global quality of life, function and symptom scores by treatment group, measured during follow‐up
**Fig. S1.** Mean unadjusted scores for global quality of life and function during follow‐up by treatment group
**Fig. S2.** Mean unadjusted scores for symptom scales during follow‐up by treatment group
**Fig. S3.** Kaplan–Meier estimates of overall and disease‐free survival by treatment allocation in the intention‐to‐treat population
