# Supplementary material for: Adjuvant statin therapy for oesophageal adenocarcinoma: the STAT‐ROC feasibility study
Source: BJS Open. 2019 Dec 14;4(1):59–70. doi: 10.1002/bjs5.50239 (PMC6996637; doi:10.1002/bjs5.50239)
Supplement: Supplementary file 1 — Table S1. Eligibility criteria Table S2. Reasons for withdrawal of treatment Table S3. Withdrawal rates for recruited participants, stratified by follow‐up period Table S4. Comparison of demographic and clinical variables between non‐randomized and randomized patient populations Table S5. Adverse events stratified by worst grade experienced and treatment group Table S6. Global quality of life, function and symptom scores by treatment group, measured during follow‐up Fig. S1. Mean unadjusted scores for global quality of life and function during follow‐up by treatment group Fig. S2. Mean unadjusted scores for symptom scales during follow‐up by treatment group Fig. S3. Kaplan–Meier estimates of overall and disease‐free survival by treatment allocation in the intention‐to‐treat population [file BJS5-4-59-s001.docx]

**BJS5_50239**

**Adjuvant statin therapy for oesophageal adenocarcinoma: the STAT-ROC feasibility study**

**L. Alexandre, A. B. Clark, S. Walton, M. P. Lewis, B. Kumar, E. C. Cheong, H. Warren, S. S. Kadirkamanathan, S. L. Parsons, S. M. Dresner, E. Sims, M. Jones, M. Hammond, M. Flather, Y. K. Loke, A. M. Swart and A. R. Hart**

**Table S1** Eligibility criteria

| Inclusion criteria. Participants were required to fulfil all of the following criteria: |
| --- |
| 1. Participant was willing and able to give informed consent for participation in the trial. |
| 1. Male or female, aged 18 years or above. |
| 1. Diagnosed with oesophageal adenocarcinoma (including adenocarcinoma of the gastro-oesophageal junction [Siewert I/II lesions]) confirmed with both endoscopy and histology. |
| 1. Due to undergo potentially curative surgery with either an oesophagectomy, oesophago-gastrectomy or extended total gastrectomy and survive to discharge from hospital following their operation. |
| Exclusion criteria – patients fulfilling any of the following criteria were excluded: |
| 1. Currently prescribed a statin as part of their routine clinical care. |
| 1. Were due to be prescribed a statin as part of their routine clinical care. Applicable to a participant who has agreed to statin therapy as recommended by their general practitioner (GP) for the primary or secondary prevention of cardiovascular disease. NB: patients who qualified for a statin but who choose not to be prescribed one for primary or secondary prevention of cardiovascular disease were still potentially eligible for this study. |
| 1. Hypersensitivity to simvastatin. |
| Active liver disease or unexplained persistent elevations of serum transaminases (> 3x ULN). |
| Severe renal insufficiency (estimated glomerular filtration rate [eGFR] less than 30 mL/minute/1.73 m^2^). |
| Creatine kinase (CK) > 5x ULN. |
| Female participants who were pregnant, lactating or planning pregnancy during the course of the trial. |
| Concomitant drug prescription of potent CYP3A4 inhibitors planned for greater than 1 month during the study period (e.g. itraconazole, ketoconazole, fluconazole, posaconazole, HIV protease inhibitors [e.g. nelfinavir], erythromycin, clarithromycin, telithromycin and nefazodone). |
| Concomitant drug prescription planned for greater than 1 month during the study period of amiodarone, verapamil, diltiazem, amlodipine, ciclosporin, danazol or gemfibrozil. |
| Acute porphyria. |

**Table S2** Reasons for withdrawal of treatment

| Number withdrawn | Proportion | Reason |
| --- | --- | --- |
| 4 | 12.5% | Difficulty swallowing trial medication |
| 1 | 3.1% | Withdrew consent (1 for treatment only) |
| 1 | 3.1% | General practitioner prescribed statin during follow-up |
| 1 | 3.1% | Adverse event (transaminitis) |
| Total: 7 | 21.9% |  |

**Table S3** Withdrawal rates for recruited participants, stratified by follow-up period

| Follow-up | Person-years | Number withdrawn | Rate (95% CI) |
| --- | --- | --- | --- |
| 0-3 months | 6.8 | 5 | 0.74 (0.31-1.77) |
| 3-6 months | 5.5 | 2 | 0.36 (0.09-1.46) |
| 6-9 months | 4.2 | 0 | 0 |
| 9-12 months | 2.8 | 0 | 0 |
| Overall | 19.3 | 7 | 0.36 (0.17-0.76) |

Rates represent the annual rate of withdrawal

**Table S4** Comparison of demographic and clinical variables between non-randomized and randomized patient populations

|  | non-randomised (n=88) | randomised (n=32) |  |
| --- | --- | --- | --- |
| Variable |  |  | p-value |
| Age at diagnosis, years | 67.8 (8.9) | 64.0 (10.8) | 0.059 |
| Gender |  |  |  |
| Male | 77 (88.5) | 25 (78.1) | 0.151 |
| Female | 10 (11.5) | 7 (21.9) |  |
| Smoking status |  |  |  |
| Current | 13 (16.5) | 4 (12.5) | 0.518 |
| Past | 42 (53.2) | 21 (65.6) |  |
| Never | 24 (30.4) | 7 (21.9) |  |
| Body mass index (kg/m^2^) | 27.6 (5.2) | 26.4 (4.4) | 0.245 |
| Comorbid conditions |  |  |  |
| Cardiovascular | 25 (28.4) | 1 (3.1) | 0.002 |
| Diabetes | 18 (22.5) | 0 | 0.002 |
| Peri-operative aspirin use | 15 (17.9) | 0 | 0.010 |
| Performance status |  |  |  |
| 0 | 53 (63.9) | 28 (87.5) | 0.023 |
| 1 | 27 (32.5) | 3 (9.4) |  |
| 2 | 3 (3.6) | 1 (3.1) |  |
| Unknown^1^ | 5 (5.7) | 0 |  |
| Tumour site |  |  |  |
| Oesophageal | 42 (50.0) | 12 (37.5) | 0.436 |
| Siewert I | 11 (13.1) | 6 (18.8) |  |
| Siewert II | 31 (36.9) | 14 (43.8) |  |
| Unknown | 4 (4.5) | 0 |  |
| Tumour grade |  |  |  |
| G1 | 1 (1.3) | 0 | 0.489 |
| G2 | 41 (51.9) | 13 (46.4) |  |
| G3 | 37 (46.8) | 14 (50) |  |
| G4 | 0 | 1 (3.6) |  |
| Unknown^1^ | 9 (10.2) | 4 (12.5) |  |
| Clinical T stage |  |  |  |
| 1 | 3 (3.5) | 0 | 0.064 |
| 2 | 15 (17.9) | 1 (3.1) |  |
| 3 | 63 (75.0) | 28 (87.5) |  |
| 4 | 3 (3.6) | 3 (9.4) |  |
| Unknown^1^ | 4 (4.5) | 0 |  |
| Clinical N stage |  |  |  |
| 0 | 38 (45.8) | 7 (21.9) | 0.067 |
| 1 | 22 (26.5) | 15 (46.9) |  |
| 2 | 16 (19.3) | 8 (25.0) |  |
| 3 | 7 (8.4) | 2 (6.3) |  |
| Unknown^1^ | 5 (5.7) | 0 |  |
| Neoadjuvant chemotherapy |  |  |  |
| Yes | 66 (76.7) | 30 (93.8) | 0.036 |
| No | 20 (23.3) | 2 (6.3) |  |
| Unknown^1^ | 2 (2.3) | 0 |  |
| Pre-operative radiotherapy |  |  |  |
| Yes | 5 (6.0) | 1 (3.1) | 0.464 |
| No | 78 (94) | 31 (97.9) |  |
| Unknown^1^ | 5 (5.7) | 0 |  |

Abbreviations: G, tumour grade; kg, kilograms; m, meters; SD, standard deviation

Values are frequencies (%) or means (SD) unless otherwise specified.

^1^Percentages presented for unknown categories reflect overall proportion of missing data for the relevant covariate; while percentages presented for known categories refer to complete data only.

**Table S5** Adverse events stratified by worst grade experienced and treatment group

Data are n for the worst grade experienced for each adverse event recorded.

CTCAE, Common Terminology Criteria for Adverse Events

**Table S6** Global quality of life, function and symptom scores by treatment group, measured during follow-up

| **Quality of life measured at 3 months** | | |  |  |  |  |
| --- | --- | --- | --- | --- | --- | --- |
|  | Placebo | | Simvastatin | | Difference at 3 months | |
|  | n | mean (SD) | n | mean (SD) | n | adjusted mean (95% CI) |
| Global QOL | 14 | 61.9 (20.3) | 15 | 63.5 (16.9) | 28 | 0.43 (-0.06 to 0.91) |
| QLQ-C30 function scores |  |  |  |  |  |  |
| Role | 14 | 65.5 (36.1) | 15 | 70.8 (30.1) | 29 | 0.53 (-0.09 to 1.14) |
| Emotional | 14 | 76.8 (24.9) | 15 | 78.0 (19.1) | 28 | 0.51 (0.27 to 0.75) |
| Cognitive | 14 | 76.2 (33.1) | 15 | 80.2 (20.4) | 28 | 0.70 (0.27 to 1.13) |
| Social | 14 | 67.9 (33.0) | 15 | 67.7 (22.3) | 28 | 0.31 (-0.05 to 0.66) |
| Physical | 14 | 76.2 (18.4) | 15 | 79.6 (18.9) | 29 | 0.37 (-0.15 to 0.90) |
| OG25 symptom scales |  |  |  |  |  |  |
| Dysphagia | 14 | 12.7 (14.4) | 15 | 17.0 (25.8) | 28 | 0.10 (-0.17 to 0.36) |
| Eating restrictions | 14 | 34.5 (24.6) | 15 | 37.4 (25.2) | 28 | 0.13 (-0.19 to 0.44) |
| Reflux | 14 | 29.8 (30.8) | 15 | 24.4 (22.6) | 28 | 0.45 (0.04 to 0.86) |
|  |  |  |  |  |  |  |
| **Quality of life measured at 6 months** | | |  |  |  |  |
|  | Placebo | | Simvastatin | | Difference at 6 months | |
|  | n | mean (SD) | n | mean (SD) | n | adjusted mean (95% CI) |
| Global QOL | 12 | 75.0 (15.9) | 13 | 71.8 (18.2) | 23 | 0.30 (-0.18 to 0.77) |
| QLQ-C30 function scores |  |  |  |  |  |  |
| Role | 12 | 86.1 (15.6) | 13 | 76.9 (28.5) | 24 | 0.70 (0.12 to 1.28) |
| Emotional | 12 | 88.2 (17.2) | 13 | 78.8 (24.2) | 23 | 0.57 (0.38 to 0.77) |
| Cognitive | 12 | 84.7 (19.4) | 13 | 74.4 (30.1) | 23 | 0.97 (0.64 to 1.31) |
| Social | 12 | 91.7 (15.1) | 13 | 74.4 (33.1) | 23 | 0.42 (0.11 to 0.74) |
| Physical | 12 | 88.9 (15.7) | 13 | 78.9 (22.2) | 25 | 1.25 (0.55 to 1.95) |
| OG25 symptom scales |  |  |  |  |  |  |
| Dysphagia | 12 | 6.5 (11.1) | 14 | 8.7 (12.5) | 25 | 0.02 (-0.21 to 0.25) |
| Eating restrictions | 12 | 24.3 (22.0) | 14 | 28.6 (20.6) | 25 | 0.28 (-0.06 to 0.62) |
| Reflux | 12 | 23.6 (25.1) | 14 | 13.1 (23.7) | 25 | 0.44 (0.02 to 0.87) |
|  |  |  |  |  |  |  |
| **Quality of life measured at 9 months** | | |  |  |  |  |
|  | Placebo | | Simvastatin | | Difference at 9 months | |
|  | n | mean (SD) | n | mean (SD) | n | adjusted mean (95% CI) |
| Global QOL | 8 | 75.0 (10.9) | 12 | 75.7 (12.5) | 18 | 0.12 (-0.31 to 0.56) |
| QLQ-C30 function scores |  |  |  |  |  |  |
| Role | 8 | 87.5 (19.4) | 12 | 81.9 (25.1) | 19 | 0.77 (0.15 to 1.39) |
| Emotional | 8 | 84.4 (22.5) | 12 | 78.5 (22.0) | 18 | 0.50 (0.31 to 0.69) |
| Cognitive | 8 | 87.5 (19.4) | 12 | 81.9 (21.9) | 18 | 0.41 (-0.02 to 0.84) |
| Social | 8 | 93.8 (17.7) | 12 | 81.9 (26.1) | 18 | 0.05 (-0.38 to 0.47) |
| Physical | 8 | 93.3 (6.2) | 12 | 84.4 (15.4) | 19 | 0.67 (0.15 to 1.20) |
| OG25 symptom scales |  |  |  |  |  |  |
| Dysphagia | 7 | 7.9 (12.4) | 12 | 7.4 (10.9) | 18 | 0.07 (-0.17 to 0.32) |
| Eating restrictions | 7 | 16.7 (17.3) | 12 | 25.7 (19.6) | 18 | 0.21 (-0.15 to 0.55) |
| Reflux | 7 | 16.7 (16.7) | 12 | 23.6 (21.9) | 18 | 0.53 (0.22 to 0.83) |

| **Quality of life measured at 12 months** | | |  |  |  |  |
| --- | --- | --- | --- | --- | --- | --- |
|  | Placebo | | Simvastatin | | Difference at 12 months | |
|  | n | mean (SD) | n | mean (SD) | n | adjusted mean (95% CI) |
| Global QOL | 4 | 83.3 (0) | 6 | 75 (13.9) | 9 | 0.20 (-0.81 to 1.22) |
| QLQ-C30 function scores |  |  |  |  |  |  |
| Role | 4 | 87.5 (8.3) | 6 | 77.8 (25.1) | 9 | 1.64 (0.72 to 2.57) |
| Emotional | 4 | 97.9 (4.2) | 6 | 75.0 (19.0) | 9 | 0.45 (0.14 to 0.75) |
| Cognitive | 4 | 95.8 (8.3) | 6 | 77.8 (31.0) | 9 | 1.40 (0.81 to 1.99) |
| Social | 4 | 100 (0) | 6 | 83.3 (18.3) | 9 | 0.26 (-0.09 to 0.62) |
| Physical | 4 | 95 (6.3) | 6 | 81.1 (22.9) | 9 | 6.17 (2.53 to 9.80) |
| OG25 symptom scales |  |  |  |  |  |  |
| Dysphagia | 4 | 2.8 (5.6) | 6 | 1.9 (4.5) | 9 | 0.28 (-0.30 to 0.86) |
| Eating restrictions | 4 | 6.3 (8.0) | 6 | 22.2 (16.4) | 9 | 0.08 (-0.77 to 0.94) |
| Reflux | 4 | 12.5 (16.0) | 6 | 19.4 (19.5) | 9 | -0.71 (-2.33 to 0.90) |

Global quality of life and functional scales: high score suggests a high level of functioning. Symptom scales: high score suggest worse symptoms. Difference in mean scores adjusted for values at screening by ANCOVA. For global quality of life and functional scales a positive difference implies that patients on Simvastatin have less deterioration than patients on placebo. For symptom scales a positive difference implies that patients on Simvastatin have more deterioration than patients on placebo. For adjusted difference, n is the number of observations included in the model.

**Fig. S1** Mean unadjusted scores for global quality of life and function during follow-up by treatment group


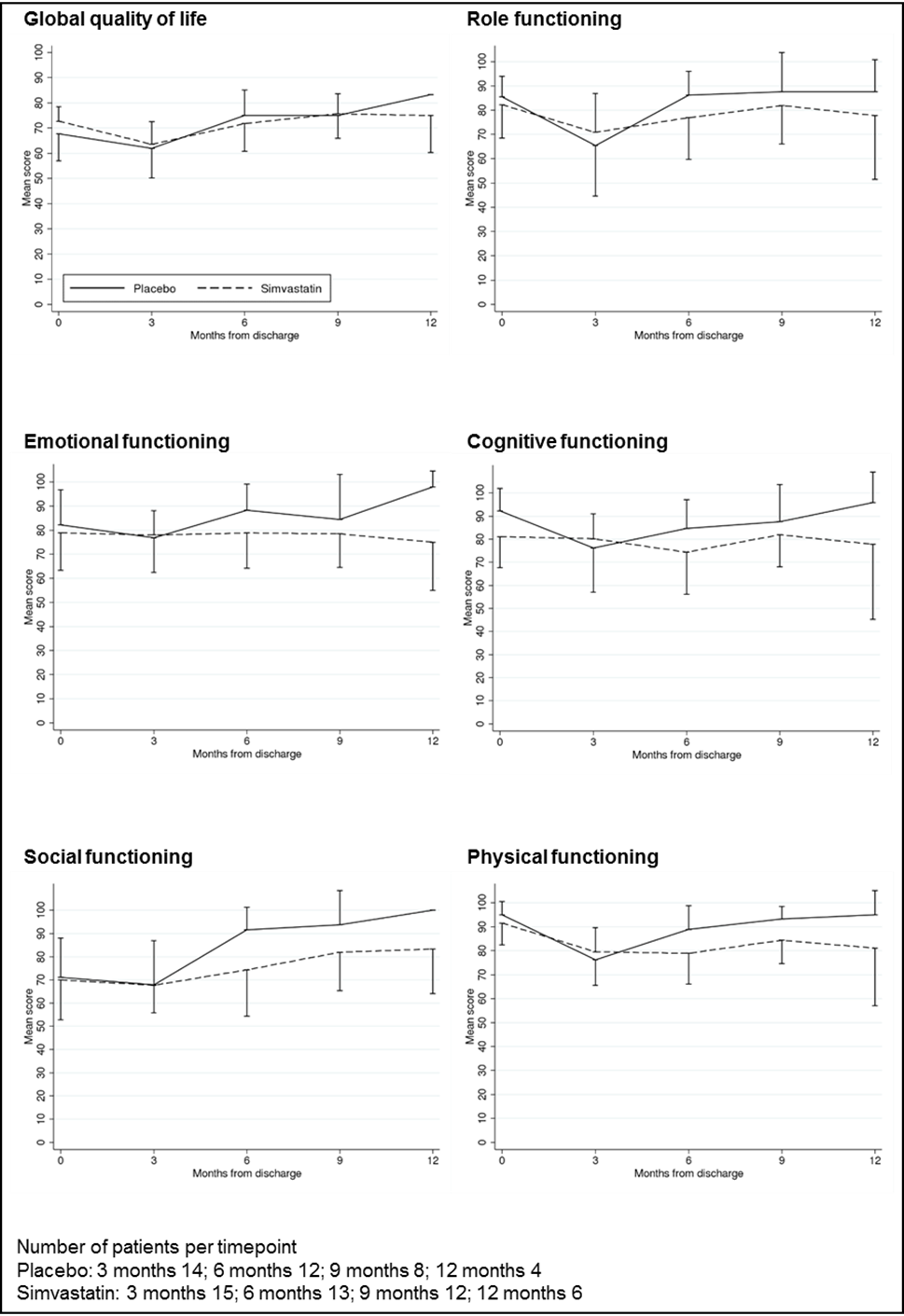


Higher scores suggest better quality of life and functioning. Half error bars span from the mean to the upper limit or lower limit of the 95% confidence interval.


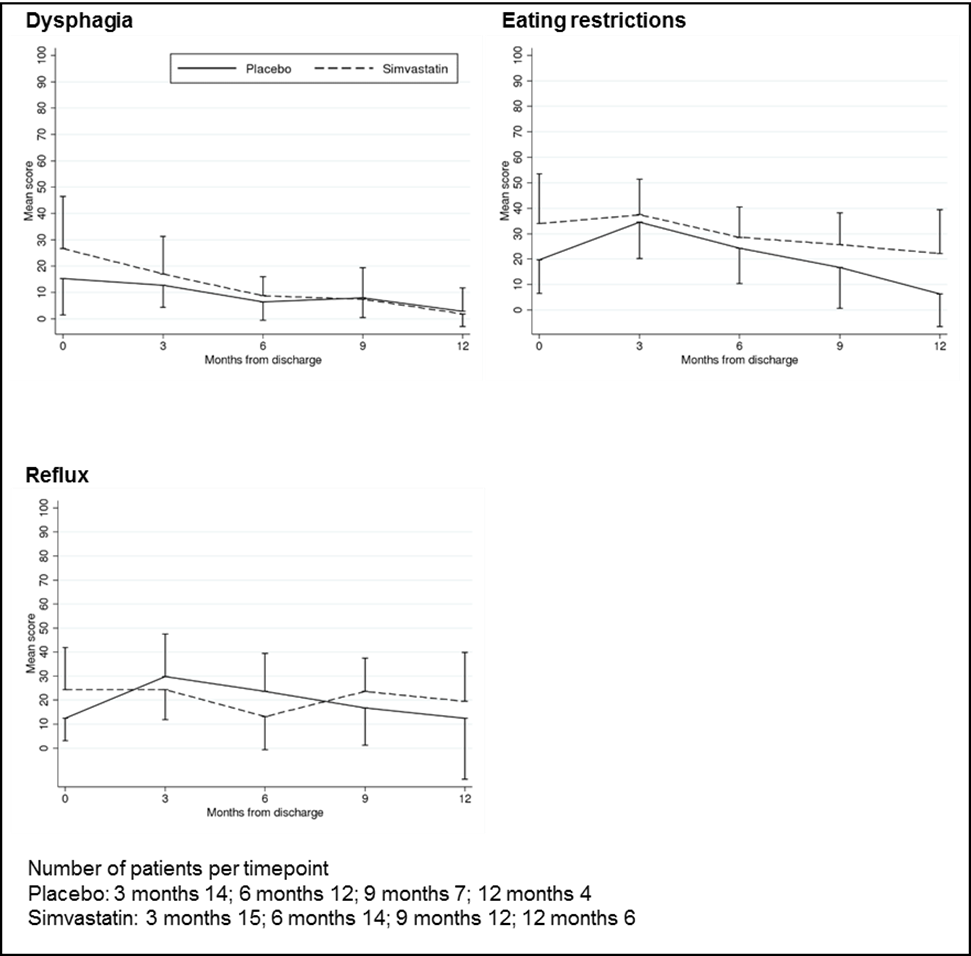
**Fig. S2** Mean unadjusted scores for symptom scales during follow-up by treatment group

Higher scores suggest worse symptoms. Half error bars span from the mean to the upper limit or lower limit of the 95% confidence interval.


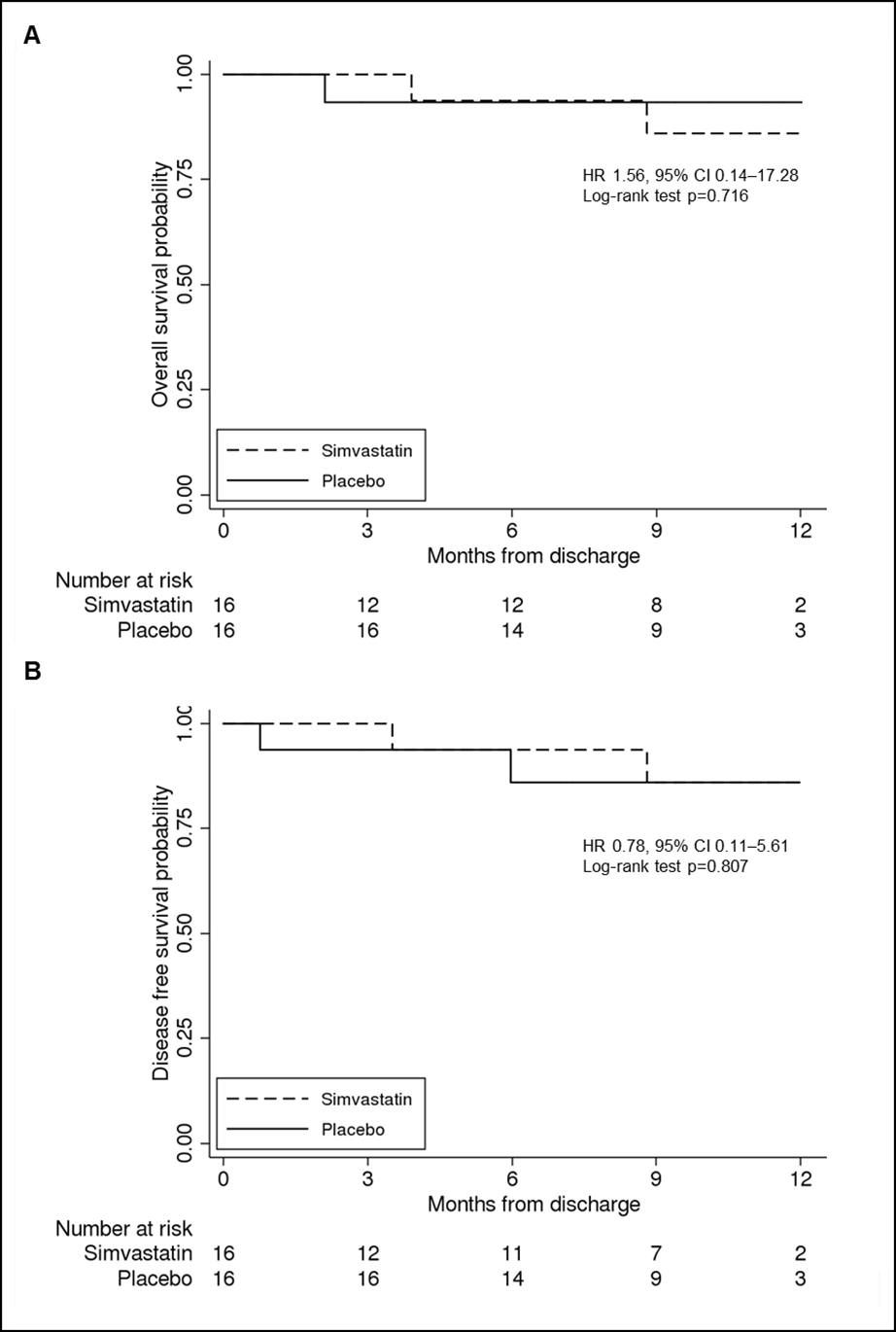
**Fig. S3** Kaplan–Meier estimates of overall and disease-free survival by treatment allocation in the intention-to-treat population

Hazard ratios and 95% confidence intervals estimated with Cox proportional hazards regression.
